# The nonstructural p17 protein of a fusogenic bat-borne reovirus regulates viral replication in virus species- and host-specific manners

**DOI:** 10.1371/journal.ppat.1010553

**Published:** 2022-06-02

**Authors:** Ryotaro Nouda, Takahiro Kawagishi, Yuta Kanai, Masayuki Shimojima, Masayuki Saijo, Yoshiharu Matsuura, Takeshi Kobayashi

**Affiliations:** 1 Department of Virology, Research Institute for Microbial Diseases, Osaka University, Suita, Osaka, Japan; 2 Special Pathogens Laboratory, Department of Virology I, National Institute of Infectious Diseases, Musashimurayama, Tokyo, Japan; 3 Laboratory of Virus Control, Research Institute for Microbial Diseases, Osaka University, Suita, Osaka, Japan; 4 Center for Infectious Disease Education and Research, Osaka University, Suita, Osaka, Japan; Washington University in Saint Louis, UNITED STATES

## Abstract

Nelson Bay orthoreovirus (NBV), a member of the family *Reoviridae*, genus *Orthoreovirus*, is a bat-borne virus that causes respiratory diseases in humans. NBV encodes two unique nonstructural proteins, fusion-associated small transmembrane (FAST) protein and p17 protein, in the S1 gene segment. FAST induces cell–cell fusion between infected cells and neighboring cells and the fusogenic activity is required for efficient viral replication. However, the function of p17 in the virus cycle is not fully understood. Here, various p17 mutant viruses including p17-deficient viruses were generated by a reverse genetics system for NBV. The results demonstrated that p17 is not essential for viral replication and does not play an important role in viral pathogenesis. On the other hand, NBV p17 regulated viral replication in a bat cell line but not in other human and animal cell lines. Nuclear localization of p17 is associated with the regulation of NBV replication in bat cells. We also found that p17 dramatically enhances the cell–cell fusion activity of NBV FAST protein for efficient replication in bat cells. Furthermore, we found that a protein homologue of NBV p17 from another bat-borne orthoreovirus, but not those of avian orthoreovirus or baboon orthoreovirus, also supported efficient viral replication in bat cells using a p17-deficient virus-based complementation approach. These results provide critical insights into the functioning of the unique replication machinery of bat-borne viruses in their natural hosts.

## Author summary

Bat-borne viruses including the severe acute respiratory syndrome coronavirus and Nipah virus generally cause highly pathogenic diseases in humans but not in their bat reservoirs. Nelson Bay orthoreovirus (NBV), a bat-borne virus associated with acute respiratory tract infections in humans, possesses two unique nonstructural proteins, FAST and p17. FAST enhances viral replication through its cell–cell fusion activity, while the function of p17 in the viral life cycle is poorly understood. In this study, we show that p17 is non-essential for viral replication in several human and animal cell lines and does not play a critical role in pathogenesis *in vivo*. However, p17 localizes to the nucleus and regulates viral replication specifically in cells derived from bats by enhancing the cell–cell fusion activity of FAST in a host-specific manner. Furthermore, the expression of NBV p17 or an NBV p17 homologue from another bat-borne orthoreovirus enhanced the replication of an NBV mutant deficient in p17 in bat cells, suggesting that the function of p17 is virus species-specific. These findings will contribute to our understanding of how the replication of viruses is regulated in their natural reservoirs.

## Introduction

Emerging viruses that spillover from their natural reservoirs are a serious threat to public health [1,2]. These viruses can cause lethal disease in humans without affecting the health of their natural host. For example, avian influenza virus H5N1 can cause severe respiratory disease with a high mortality rate in humans, but not in duck species [3], and hantavirus can cause hemorrhagic fever in humans, but is asymptomatic when it infects rodents [4,5]. Bats are thought to be the natural reservoirs for a number of deadly zoonotic viruses, including severe acute respiratory syndrome (SARS) coronaviruses, Ebola virus, and Nipah virus [6–8], and are regarded as the most likely natural host for the COVID-19 infectious agent, SARS-CoV-2 [9–11]. While no mortality or morbidity has been observed in bats experimentally infected with Hendra virus, Nipah virus, Marburg virus, Ebola virus, or SARS-like coronaviruses [12–15], the precise mechanisms that allow these viruses to circumvent pathogenesis in bats remain to be elucidated. These observations suggest that bat-borne viruses build symbiotic relationships in their reservoir hosts to regulate viral replication [8]. Such relationships between virus and host are thought to be established by complex mechanisms regulated by antiviral immune defenses and viral factors. Constitutive expression of interferon (IFN)-α has been observed in unstimulated bat tissue and cultured cells, and it is possible that constitutively expressed IFN and IFN-stimulated genes control viral replication in bat species [16]. It has similarly been reported that stimulator of IFN genes (STING), which is an important innate immune response protein involved in DNA sensing, and NLR family pyrin domain containing (NLRP) 3, which activates inflammatory mediators, are dampened in bats [17,18], contributing to a reduced inflammatory response and disease tolerance. However, although much is known about antiviral immune defenses in bats, the viral factors responsible for the control of replication of bat-borne viruses in their natural reservoirs remain largely unknown.

Nelson Bay orthoreovirus (NBV), a member of the genus *Orthoreovirus* belonging to the family *Reoviridae*, was first isolated in a fruit bat in Australia in 1968 [19]. Several NBV strains have since been isolated from different bat species [20–23] and, therefore, bats have been suggested as the natural reservoir for NBVs. Recently, NBVs have been isolated from patients with acute respiratory infection in several Asian countries [24–26]. In Japan, a pathogenic NBV Miyazaki-Bali/2007 (MB) strain was isolated from a Japanese patient who traveled to Bali, Indonesia [27]. These findings suggest that NBV has evolved to cross the species barrier from bats to humans. Members of the genus *Orthoreovirus* are divided into fusogenic and nonfusogenic subgroups, based on their ability to induce cell–cell fusion [28]. The orthoreovirus genome consists of ten segments of double-stranded RNA (dsRNA) (L1–L3, M1–M3, and S1–S4 gene segments) and some fusogenic reovirus genomes, including avian orthoreovirus (ARV), baboon orthoreovirus (BRV), Broome orthoreovirus (BroV), and NBV, encode two unique nonstructural proteins, the fusion-associated small transmembrane (FAST) protein and p17, on the S1 or S4 gene segment [29–32]. FAST proteins are small fusogenic proteins of approximately 10–22 kDa that can induce cell–cell fusion between infected-cells and neighboring cells [32,33]. FAST proteins are composed of three functional domains associated with fusion activity: an N-terminal ectodomain, a transmembrane domain, and a C-terminal cytoplasmic domain [34,35]. Unlike the structural proteins of enveloped viruses, nonstructural FAST proteins are dispensable for viral entry [36,37]. While the biological function of FAST proteins is poorly understood, it has been demonstrated that FAST is required for efficient viral propagation *in vitro* and plays a crucial role in pathogenesis *in vivo* [37].

Nonstructural NBV p17, ARV p17, BRV p16, and BroV p16 proteins are encoded by the second open reading frame of the fusogenic reovirus S1 or S4 gene segment [30,31,38,39]. Previous studies have revealed that ARV p17, also known as CRM1-independent nucleocytoplasmic shuttling protein, possesses a nuclear localization signal with critical C-terminal basic amino acids at residues K122 and R123 conserved among ARV and NBV strains [40]. In addition, it has been shown that ARV p17 activates the p53 signaling pathway and downregulates the PI3K/AKT/mTOR and ERK pathways [41]. The modulation of these fundamental signaling pathways results in the disruption of cellular translation, cell cycle arrest, and the formation of autophagosomes, contributing to efficient viral replication [41–44]. However, the biological functions of NBV p17 in the viral life cycle, and its involvement in viral pathogenesis, remain unclear.

In this study, we examined the roles of NBV p17 in viral replication in cell lines and viral pathogenesis in a mouse model by using p17-mutant and -deficient viruses. We found that replication of p17-deficient virus was severely impaired in cell lines derived from the fruit bat *Rousettus leschenaultii*, a potential natural host for NBV. Expression of NBV p17 and BroV p16, but not other p17 homologues, rescued the cell–cell fusion activity of NBV FAST, leading to efficient replication in bat cells, suggesting that p17 regulates viral replication in virus species- and host-specific manners.

## Results

### NBV p17 is not required for viral replication *in vitro*

**Fig 1:**
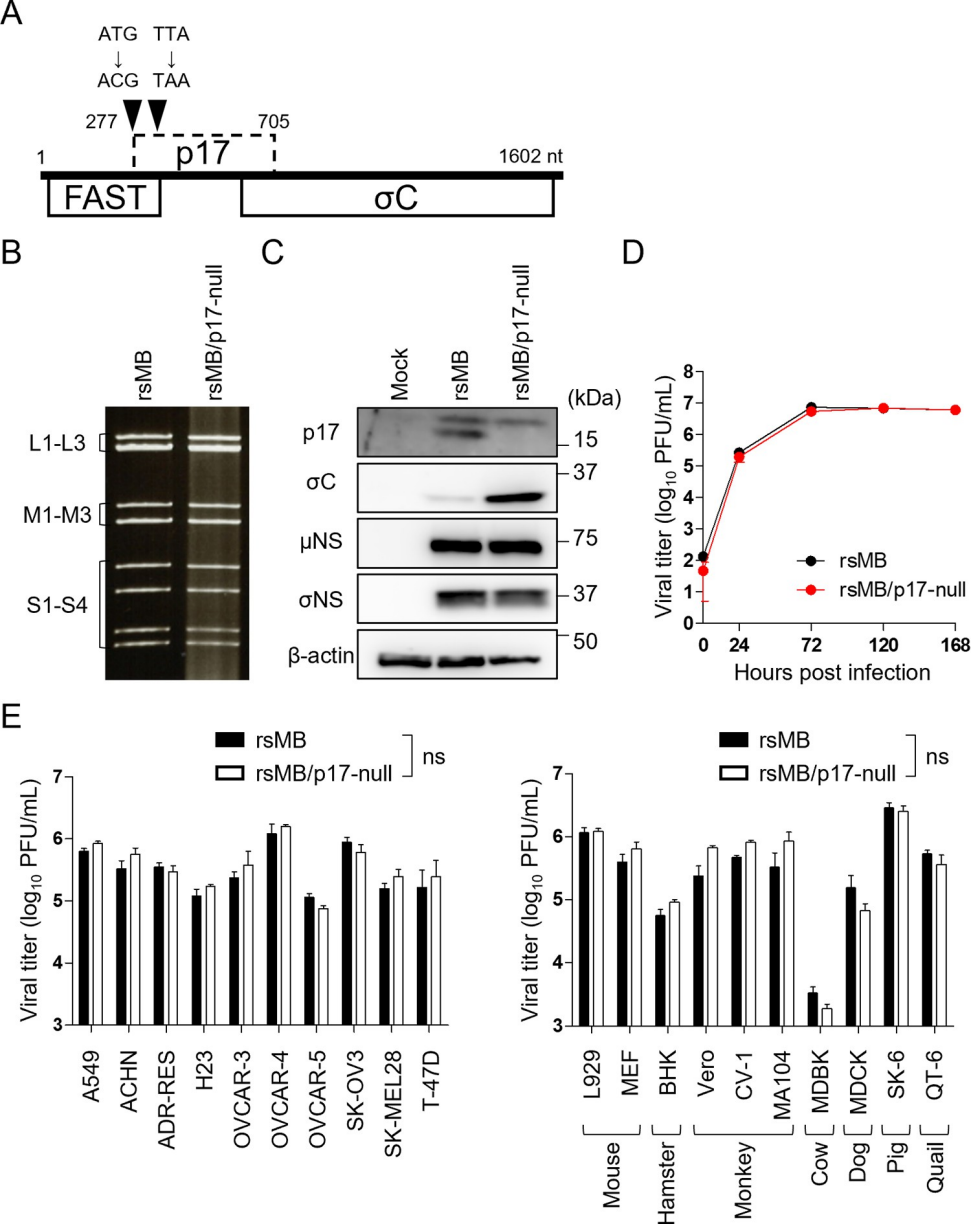
Evaluation of viral replication following wild-type and p17-null virus infection. **(A)** Schematic presentation of the S1 gene segment of rsMB and rsMB/p17-null. Three ORFs, FAST (27–314 nt), p17 (277–705 nt), and σC (572–1567 nt), are encoded on the NBV S1 gene segment. In the p17-null virus, the p17 start codon is disrupted with a ^277^ATG^279^→ACG substitution, and a stop codon introduced by a ^301^TTA^303^→TAA substitution. **(B)** The electropherotype of the genomic RNA of rsMB and rsMB/p17-null. RNA extracted from virions was electrophoresed on a 10% polyacrylamide gel. **(C)** Electrophoretic analysis of viral proteins of rsMB and rsMB/p17-null. Vero cells were infected with rsMB or rsMB/p17-null at an MOI of 5 PFU/cell and lysed at 18.5 h post-infection. Cell lysates were subjected to immunoblotting using antibodies raised against NBV p17, NBV σC, NBV μNS, NBV σNS, or β-actin. **(D)** Growth kinetics of rsMB and rsMB/p17-null in Vero cells. The cells were infected with rsMB or rsMB/p17-null at an MOI of 3 PFU/cell (n = 3). Viral titers were determined by plaque assay using L929 cells. **(E)** Viral titers of rsMB and rsMB/p17-null in human and animal cell lines. Cells were infected with rsMB or rsMB/p17-null at an MOI of 0.1 PFU/cell and collected at 72 h post-infection (n = 3). Viral titers were determined by plaque assay using L929 cells. Results are expressed as the mean of three samples. Error bars indicate standard deviations. ns, not significant (Welch’s *t*-test, p > 0.01).

To understand the function of NBV p17 during the viral life cycle, we generated a p17-deficient mutant virus, using a reverse genetics system, in the pathogenic NBV strain MB [27,45]. A viable p17-null virus (rsMB/p17-null) was rescued from cells transfected with plasmids containing cDNA from nine NBV MB gene segments and S1 cDNA featuring a disrupted p17 translational start codon (^277^ATG^279^→ACG) and a stop codon (^301^TTA^303^→TAA) (Fig 1A and S1 Table). The genomic dsRNA pattern of rsMB/p17-null showed the same pattern as that of wild-type recombinant strain MB (rsMB) by electrophoresis (Fig 1B). To examine whether p17 is expressed in cells infected with rsMB/p17-null, we confirmed viral protein expression by immunoblotting protein extracts derived from infected cells. We found that while p17 expression was not detectable in cells infected with p17-null virus, the expression of NBV proteins, μNS (a component of the viral inclusion body where virus replication occurs) and σNS (a component of viral inclusion body) encoded by M3 and S3 segments, respectively, remained unchanged between p17-null virus- and wild-type virus-infected cells (Fig 1C). Expression of σC (cell-attachment protein) located downstream of the p17 ORF was increased in cells infected with the p17-null virus. The result is consistent with a previous report [46]. To examine whether p17 plays a critical role in viral replication in cultured cells, wild-type and p17-null viruses were infected into various cell lines, and viral titers were determined by plaque assay. The growth kinetics of rsMB and rsMB/p17-null were similar in Vero cells (Fig 1D) and in cell lines from humans and other animal species, such as, mouse, hamster, monkey, cow, dog, pig, and quail (Fig 1E). These data indicate that p17 is not essential for viral replication and does not affect viral propagation in these human or animal cell lines.

### NBV p17-deficient virus causes lethal infection in an NBV mouse model

**Fig 2:**
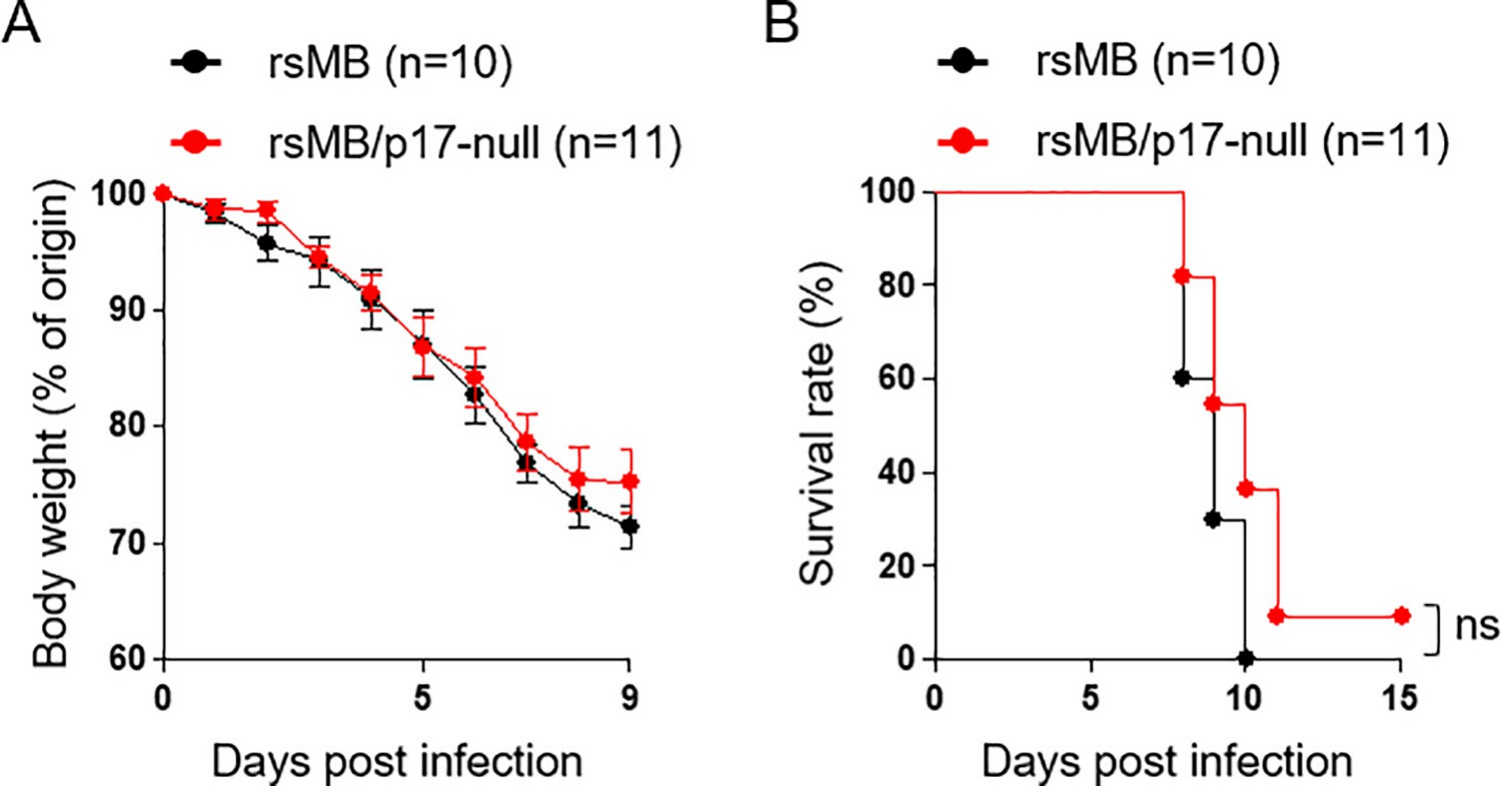
NBV p17 does not affect viral pathogenesis *in vivo*. C3H mice were intranasally infected with rsMB (n = 10) or rsMB/p17-null (n = 11). Virus suspensions (20 μL) including 3×10^5^ PFU virions were inoculated. No significant difference in body weight **(A)** or survival **(B)** was detected between groups. Error bars indicate standard deviations. ns, not significant (log rank test, p > 0.05).

In a previous study, we developed a mouse model for lethal NBV lung infection, which revealed that NBV proteins, σC and FAST, were virulence factors [37,45,47]. To understand the importance of p17 in viral pathogenesis, we intranasally inoculated 4-week-old C3H mice with either rsMB or rsMB/p17-null virus. No significant difference in body weight or survival rate was observed between mice infected with the rsMB virus and those infected with the rsMB/p17-null virus (Fig 2A and 2B), suggesting that p17 does not play an important role in viral pathogenesis in this model.

### NBV p17 regulates viral replication in the bat cell line DemKT1

**Fig 3:**
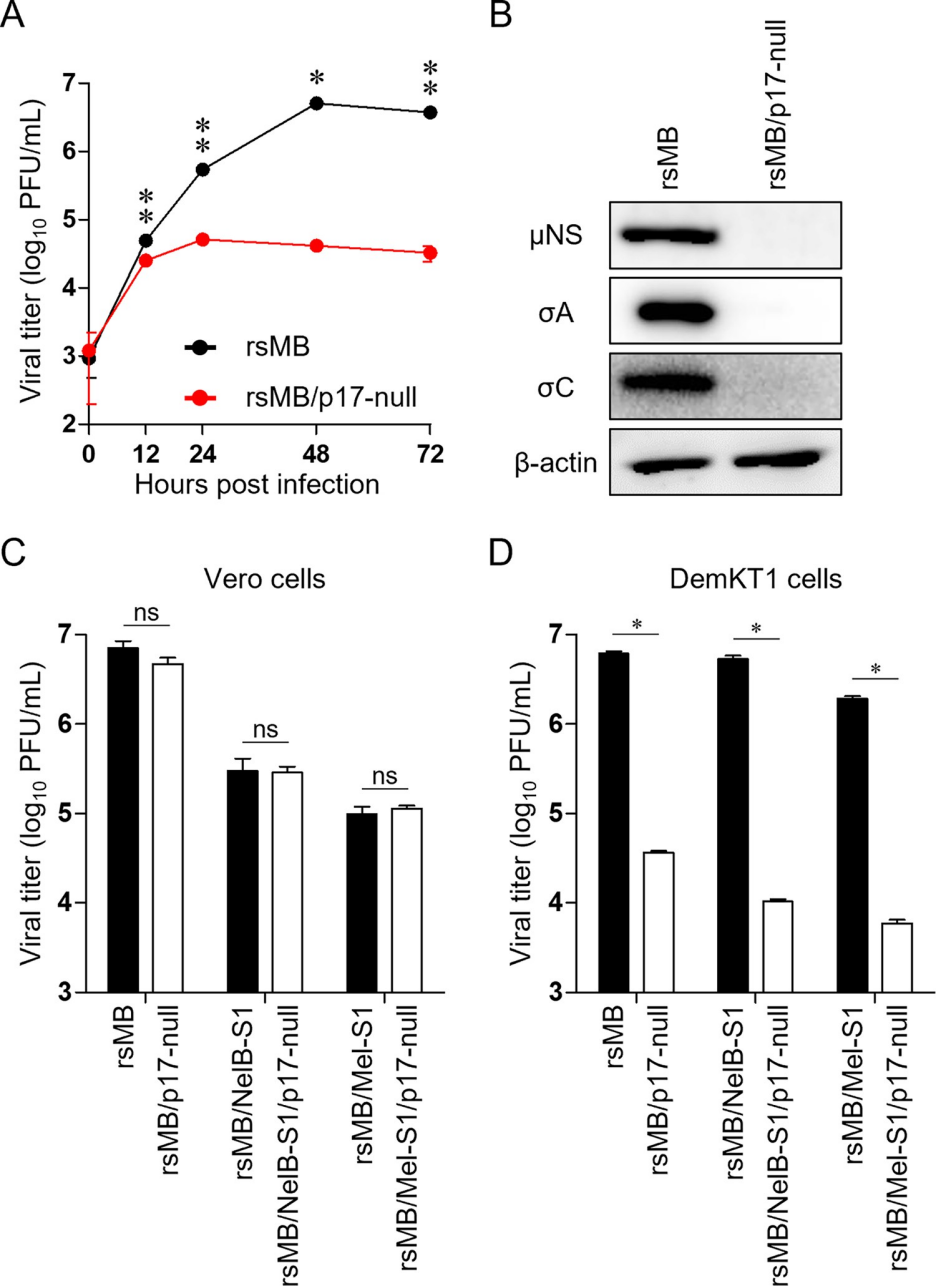
Disruption of p17 inhibits NBV replication in bat-derived DemKT1 cells. **(A)** Growth kinetics of rsMB and rsMB/p17-null in DemKT1 cells. The cells were infected with rsMB or rsMB/p17-null at an MOI of 0.1 PFU/cell (n = 3). Viral titers were determined by plaque assay using L929 cells. *p < 0.05, **p < 0.01 (Welch’s *t*-test). **(B)** Viral protein expression following rsMB and rsMB/p17-null infection. DemKT1 cells were infected with virus at an MOI of 0.1 PFU/cell and lysed at 16 h post-infection. Cell lysates were subjected to immunoblotting using antibodies raised against NBV μNS, NBV σA, NBV σC, or β-actin. **(C, D)** Replication of NBV S1 monoreassortants and their p17-deficient viruses in Vero **(C)** and DemKT1 cells **(D)**. Cells were infected at an MOI of 0.1 PFU/cell (n = 3) and plaque assay was performed at 72 h post-infection. Results are expressed as the mean of three samples. Error bars indicate standard deviations. ns, not significant; *p < 0.01 (Welch’s *t*-test).

Recent epidemiological studies have reported that NBV infects several species of bat in their natural habitat, including *Rousettus leschenaultii*, suggesting bats as the presumptive natural reservoirs of NBV [20–23,48]. Thus, we hypothesized that p17 protein could have specific functions in this natural host. To understand the importance of p17 in bats, we infected a bat cell line derived from *Rousettus leschenaultii*, DemKT1 [49], with either rsMB or rsMB/p17-null virus. Interestingly, the growth kinetics of rsMB/p17-null were significantly lower than those of wild-type rsMB (approximately 120-fold at 72 h post-infection) (Fig 3A). DemKT1 cells infected with rsMB/p17-null displayed improved cell viability compared with cells infected with rsMB (S1 Fig). The expression of several NBV proteins, including μNS, σA (inner capsid protein encoded by the S2 segment), and σC, was lower at 16 h post-infection in cells infected with the rsMB/p17-null than in those infected with wild-type rsMB (Fig 3B). Together, these data indicated that p17 plays a critical role in the regulation of viral replication in the bat DemKT1 cell line. To investigate whether the function of p17 in viral replication observed in this bat cell line is conserved among other NBV strains, we generated monoreassortant viruses containing the S1 segment from two other NBV strains, Nelson Bay (NelB), a strain isolated from bats, and Melaka (Mel), a strain isolated from human, in an otherwise MB genetic background (rsMB/NelB-S1 and rsMB/Mel-S1, respectively). p17-deficient viruses were generated in these monoreassortant strains, as described above (S1 Table), resulting in the generation of rsMB/NelB-S1/p17-null and rsMB/Mel-S1/p17-null mutants. The replication kinetics of p17-null monoreassortants were similar to those of monoreassortant viruses expressing wild-type p17 in Vero cells (Fig 3C); however, the replication kinetics were reduced 325- and 516-fold in DemKT1 cells upon infection with rsMB/Mel-S1 and rsMB/NelB-S1, respectively (Fig 3D). These results suggest that the function for NBV p17 in the regulation of viral replication in bat cells is conserved among NBV strains.

### Two p17 basic amino acids, K131 and R132, are essential for viral replication in DemKT1 cells

**Fig 4:**
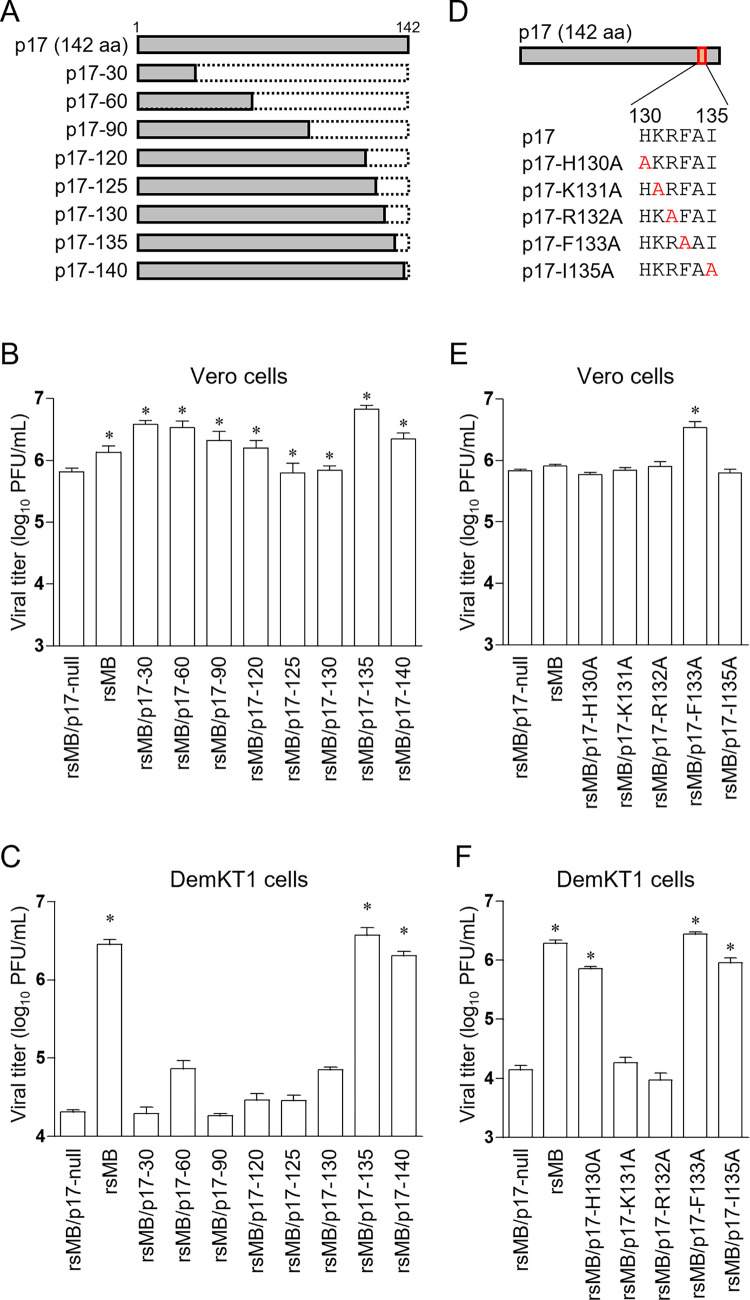
Identification of functional regions of p17 associated with viral replication in DemKT1 cells. **(A)** Schematic presentation of the p17 C-terminal truncation mutants generated in this study. **(B, C)** Replication of rsMB, rsMB/p17-null, and p17 C-terminal truncated mutants in Vero **(B)** and DemKT1 cells **(C)** infected with virus at an MOI of 0.1 PFU/cell (n = 3). Cells were collected at 72 h post-infection. Viral titers were determined by plaque assay. Results are expressed as the mean of the results of three samples. Error bars indicate standard deviations. Significant differences compared to rsMB/p17-null are indicated by asterisks. *p < 0.05 (Dunnett’s test). **(D)** Schematic presentation of alanine substitution mutants generated for this study. Each amino acid between position 130 and 135 was substituted with an alanine residue. **(E, F)** Replication of rsMB, rsMB/p17-null, and p17 alanine substitution mutant viruses in Vero **(E)** and DemKT1 cells **(F)** infected with recombinant viruses at an MOI of 0.1 PFU/cell (n = 3). Viral titers were determined by plaque assay at 72 h post-infection. Results are expressed as the mean for three samples. Error bars indicate standard deviations. Significant differences compared to rsMB/p17-null are indicated by asterisks. *p < 0.05 (Dunnett’s test).

To identify regions of p17 associated with the regulation of viral replication, we generated p17 truncation mutants lacking 112, 82, 52, 22, 17, 12, 7, or 2 amino acid residues from the C-terminus (rsMB/p17-30, rsMB/p17-60, rsMB/p17-90, rsMB/p17-120, rsMB/p17-125, rsMB/p17-130, rsMB/p17-135, and rsMB/p17-140, respectively) by the introduction of stop codons in the p17 ORF (Fig 4A). All rsMB/p17 truncation mutants replicated efficiently in Vero cells (Fig 4B), however, replication in DemKT1 cells was impaired upon infection with rsMB/p17-30, -60, -90, -120, -125, or -130, but not upon infection with rsMB/p17-135 or rsMB/p17-140 (Fig 4C). These results suggest that amino acid residues from 131 to 135 are critical for the regulation of viral replication in DemKT1 cells. To further understand the importance of these residues, alanine scanning was performed within the identified amino acids (^130^His-Lys-Arg-Phe-Ala-Ile^135^) (Fig 4D). Although the alanine substitution mutants replicated as efficiently as wild-type rsMB in Vero cells (Fig 4E), the viral titer was significantly lower in DemKT1 cells infected with rsMB/p17-K131A and rsMB/p17-R132A than in those infected with rsMB (Fig 4F). These results indicate that two basic amino acids, K131 and R132, in p17 play an important role in the regulation of NBV replication in DemKT1 cells.

### The K117, R118, K131, and R132 amino acid residues of NBV p17 are important for nuclear localization and replication in DemKT1 cells

**Fig 5:**
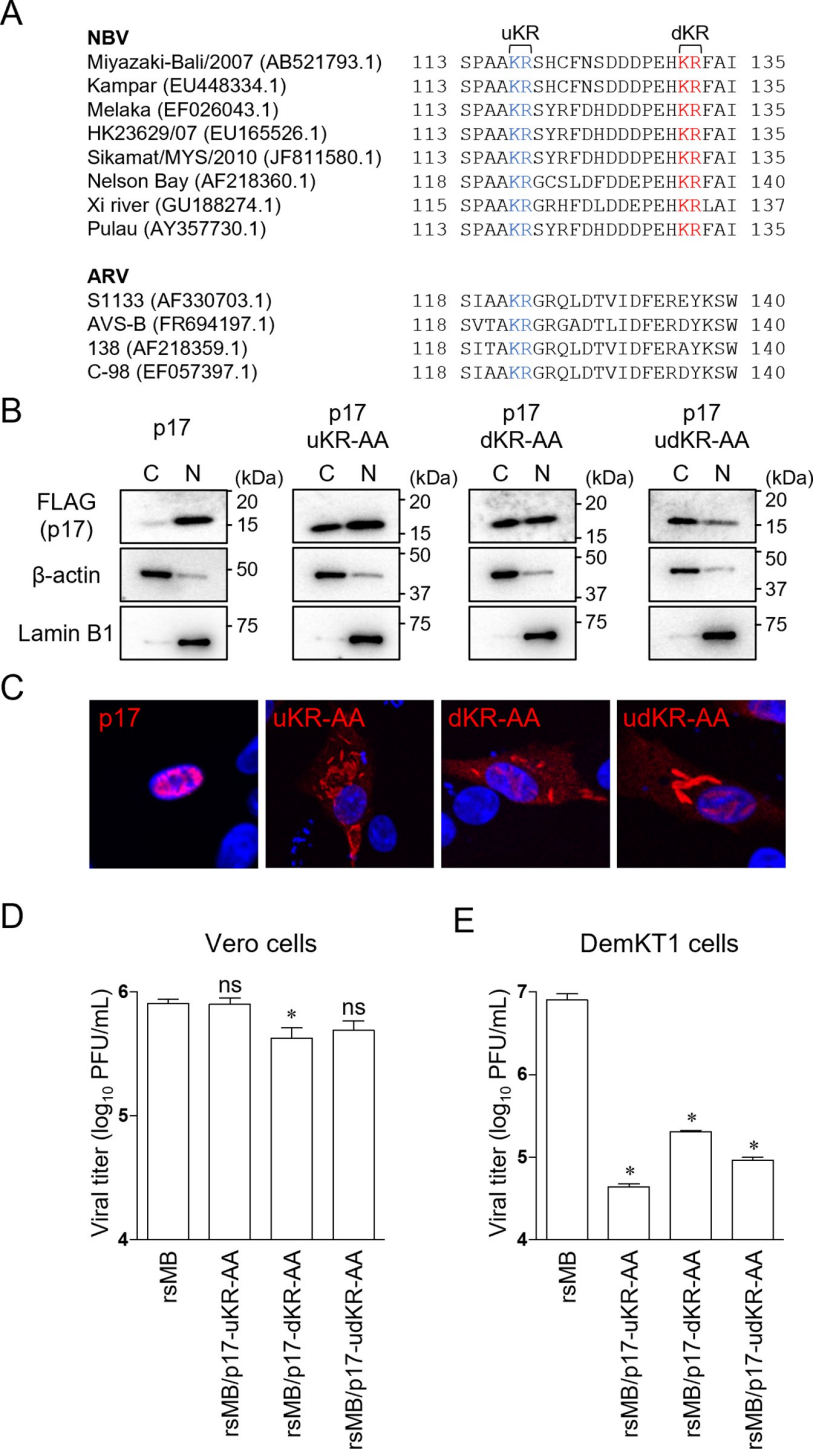
Functional analysis of two bipartite basic amino acid sequences in the p17 protein. **(A)** Conservation of basic amino acid motifs in p17 from NBV and ARV strains. The upstream basic amino acid residues (uKR) conserved among NBV and ARV strains are highlighted in blue. The downstream basic amino acid residues (dKR) conserved among NBV strains only are highlighted in red. GenBank accession numbers for each protein are in parentheses. **(B)** Subcellular localization of NBV p17 and p17 mutants in DemKT1 cells. DemKT1 cells transiently expressing FLAG epitope-tagged NBV p17 were collected at 24 h post-transfection and separated into cytoplasmic and nuclear fractions (C and N, respectively). The p17 mutant plasmids express p17 uKR-AA (K117A and R118A), dKR-AA (K131A and R132A), or udKR-AA (K117A, R118A, K131A, and R132A). **(C)** Immunostaining of NBV-p17 and its mutants in DemKT1 cells. At 25 h post-transfection, cells were stained with anti-FLAG antibody (red) and DAPI (blue). **(D, E)** Replication of wild-type and p17 mutant NBV in Vero **(D)** and DemKT1 cells **(E)** infected with each virus at an MOI of 0.1 PFU/cell (n = 3). Viral titers were determined by plaque assay at 72 h post-infection. Results are expressed as the mean of three samples. Error bars indicate standard deviations. Significant differences compared to rsMB are indicated by asterisks. ns, not significant; *p < 0.05 (Dunnett’s test).

p17 K131 and R132 are conserved in NBV strains, but not ARV strains (Fig 5A). However, a second basic amino acid motif located adjacent to K131 and R132 is conserved among both NBV and ARV strains (K117 to R118 in NBV, and K122 to R123 in ARV) (Fig 5A). It has previously been reported that ARV p17 K122A and R123A mutants impair nuclear localization [40], and we hypothesized that NBV p17 K117, R118, K131, and R132 could similarly be required for nuclear localization and the regulation of viral replication. To determine the importance of the two basic amino acid motifs in viral replication, we named the upstream K117 and R118 motif as uKR and downstream K131 and R132 motif as dKR, substituted each pair of amino acids with alanine residues in transient expression vectors, and examined the distribution of p17 in DemKT1 cells. While native p17 protein was found solely in the nucleus of transfected cells by immunoblotting (Fig 5B) and immunofluorescence analysis (Fig 5C), alanine substitution of uKR and/or dKR led to an accumulation of p17 in the cytoplasm. These data suggest that both p17 basic amino acid motifs are required for nuclear localization. To investigate the relationship between nuclear localization of p17 and viral replication in DemKT1 cells, we generated p17 mutant viruses in which uKR and/or dKR were substituted to alanine and examined the growth of these viruses using the plaque assay in Vero and DemKT1 cells. The p17 uKR and dKR mutants replicated efficiently in Vero cells (Fig 5D), while viral titers were significantly lower in DemKT1 cells (Fig 5E). These results suggest that nuclear localization of p17 is essential for efficient viral replication in DemKT1 cells.

### The transient expression of p17 complements the replication defect of rsMB/p17-null virus in DemKT1 cells

**Fig 6:**
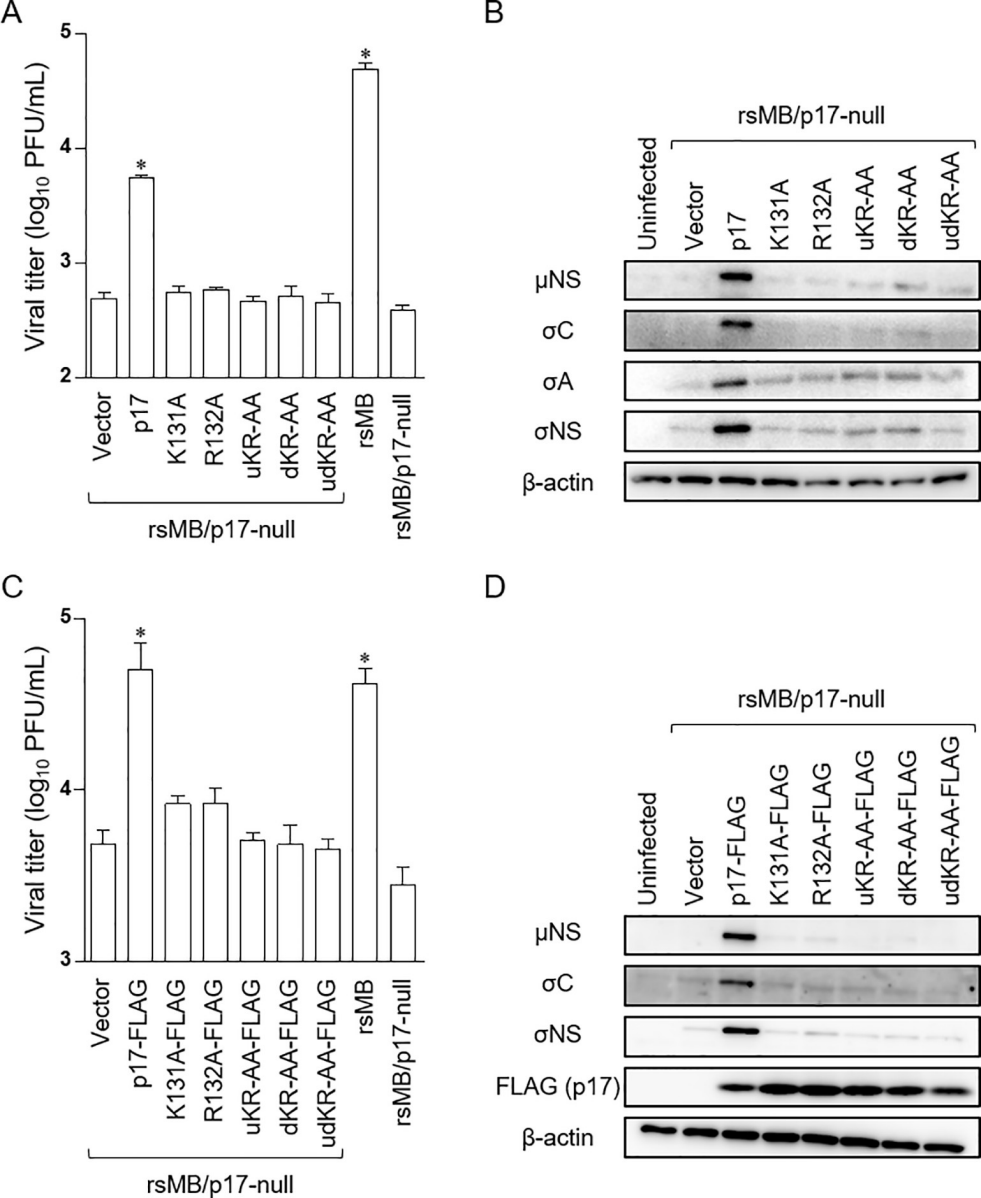
The two bipartite basic amino acid sequences in the p17 C-terminus play an important role in NBV replication in DemKT1 cells. **(A, C)** Viral titers of rsMB/p17-null in DemKT1 cells expressing wild-type and mutant p17 proteins **(A)** or FLAG-tagged wild-type and mutant p17 proteins **(C)**. Cells were infected with rsMB/p17-null at an MOI of 0.1 PFU/cell (n = 3) at 24 h post-transfection, and viral titers were determined by plaque assay at 24 h post-infection. Results are expressed as the mean of three samples. Error bars indicate standard deviations. Significant differences compared to the vector control are indicated by asterisks. *p < 0.05 (Dunnett’s test). **(B, D)** Electrophoretic analysis of viral proteins in rsMB/p17-null-infected cells expressing wild-type and mutant p17 proteins **(B)** or FLAG-tagged wild-type and mutant p17 proteins **(D)**. Wild-type or mutant p17 expression plasmids were transfected into DemKT1 cells and infected with rsMB/p17-null at an MOI of 1 PFU/cell at 10 h post-transfection. Cells were lysed at 16 h post-infection. Cell lysates were subjected to immunoblotting using antibodies raised against NBV proteins or β-actin.

p17 alanine substituted mutations in uKR and/or dKR motifs also introduced mutations in the N-terminus of the overlapping σC ORF (S1 Table). Thus, it is possible that the replication defect of p17 uKR and dKR mutants in DemKT1 cells was caused by modification of σC function. Therefore, we developed a p17 complementation system to examine whether p17 is directly responsible for efficient viral replication. DemKT1 cells were transfected with wild-type or mutant p17 expression plasmids and subsequently infected with rsMB/p17-null virus at an MOI of 1 or 0.1 PFU/cell at 10 h or 24 h post-transfection. Expression of wild-type p17 *in trans* rescued rsMB/p17-null viral growth and the expression of the viral proteins μNS, σC, σA, and σNS (Fig 6A and 6B). By contrast, all p17 constructs encoding uKR and dKR motif mutations failed to restore rsMB/p17-null virus replication (Fig 6A and 6B). We also observed that replication of the p17-null virus was restored by transient transfection with FLAG-tagged wild type p17 but not its mutants (K131A-FLAG, R132A-FLAG, uKR-AA-FLAG, dKR-AA-FLAG, and udKR-AA-FLAG) in DemKT1 cells (Fig 6C and 6D). These results confirm that nuclear localization of p17 plays a critical role in the regulation of NBV replication in DemKT1 cells.

### Expression of NBV p17 is required for induction of fusion activity of FAST in bat cell lines

**Fig 7:**
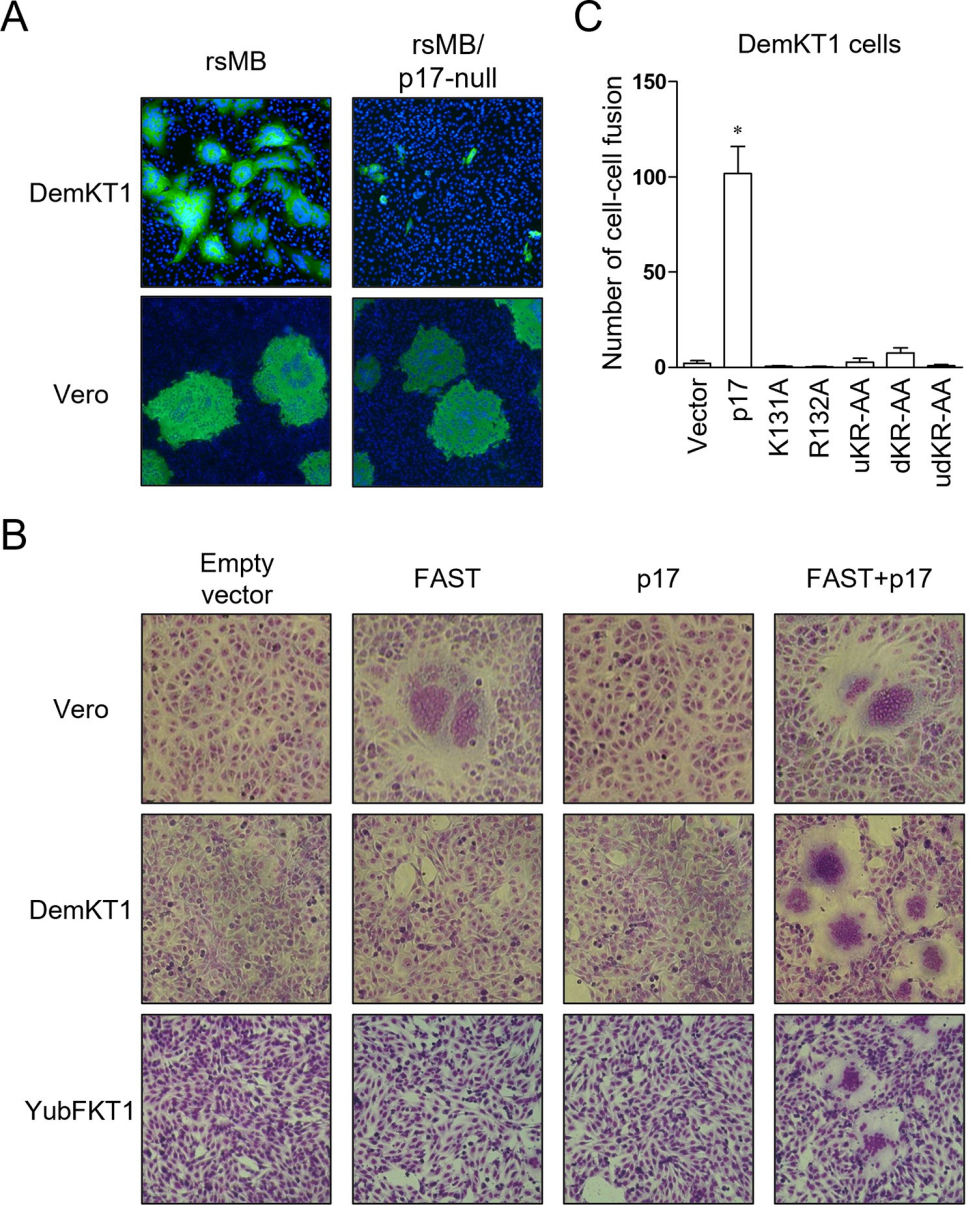
Cell–cell fusion activity of FAST is enhanced in the presence of p17 in bat cells. **(A)** Immunostaining of DemKT1 and Vero cells infected with rsMB or rsMB/p17-null at an MOI of 0.1 PFU/cell. Infected cells were stained using DAPI (blue) and an anti-NBV σC antibody (green) to observe cell–cell fusion. **(B)** Observation of cell–cell fusion activity. NBV p17 and/or FAST expression plasmids were transfected into Vero (monkey), DemKT1 (bat), and YubFKT1 (bat) cells. Transfected cells were fixed and stained with Giemsa’s Stain Solution. **(C)** The number of cell–cell fusions in DemKT1 cells expressing NBV FAST and wild-type or mutant p17s. Results are expressed as the mean of total samples. Error bars indicate standard deviations (n = 5). Significant differences compared to the vector control are indicated by asterisks. *p < 0.05 (Dunnett’s test).

Both the wild-type rsMB and rsMB/p17-null viruses encode intact FAST protein in the S1 gene segment. FAST proteins are the only known fusogenic proteins found in non-enveloped viruses and can induce cell–cell fusion in infected cells [33,50]. Recently, we demonstrated that although FAST is not essential for viral replication, it can enhance replication and pathogenesis [37]. Interestingly, in the present study, we found that the rsMB/p17-null mutant virus induced fewer cell–cell fusion events than wild-type rsMB in DemKT1 cells, while cell–cell fusion was unaffected in Vero cells (Fig 7A). These data suggest that NBV p17 may regulate FAST function during viral replication in DemKT1 cells. Therefore, we next examined the fusion activity of FAST in bat cell lines transfected with p17. In Vero cells, expression of FAST induced the formation of large multi-nuclear cells, termed syncytia, during cell–cell fusion, irrespective of co-expression of p17, (Fig 7B), consistent with previous observations [32,50]. By contrast, cell–cell fusion was induced in DemKT1 cells only by the co-expression of FAST and p17 but not by either alone (Fig 7B). To investigate whether the FAST expression level influences cell–cell fusion in transfected DemKT1 cells, an expression vector encoding FAST with two HA-tags (FAST-2HA) was transfected with/without the p17-FLAG expression plasmid. Anti-HA antibody staining showed that FAST-2HA expression dose-dependently increased with the amount of the FAST expression plasmid (0.3, 1, and 3 μg) (S2A Fig). The number of cell–cell fusions was significantly increased in cells transfected with FAST-2HA and p17-FLAG vectors but not in cells transfected with the FAST-2HA vector alone (1 and 3 μg) (S2B Fig). These results suggest that expression of p17 enhances the cell–cell fusion activity of FAST in DemKT1 cells. This induction of cell–cell fusion by co-expression of FAST with p17 in DemKT1 cells, was also observed in another bat cell line, YubFKT1, derived from the fruit bat *Miniopterus fuliginosus* (Figs 7B and S3), suggesting that the enhancement of fusion activity of FAST by p17 is specific to bat cell lines. To further confirm the role of p17 in the regulation of FAST fusion activity, we examined FAST-mediated cell–cell fusion activity in the presence of either wild-type or mutant p17. Expression of wild-type but not of either of the p17 mutants lacking nuclear localization activity induced cell–cell fusion in DemKT1 cells (Fig 7C). We also observed that cell–cell fusion was clearly induced by transient transfection of FLAG-tagged wild-type p17 but not its mutants in DemKT1 cells (S4A and S4B Fig). These results suggest that nuclear localization of p17 is required for induction of fusion activity of FAST in bat cells.

### Enhancement of FAST function by p17 is required for viral replication in DemKT1 cells

**Fig 8:**
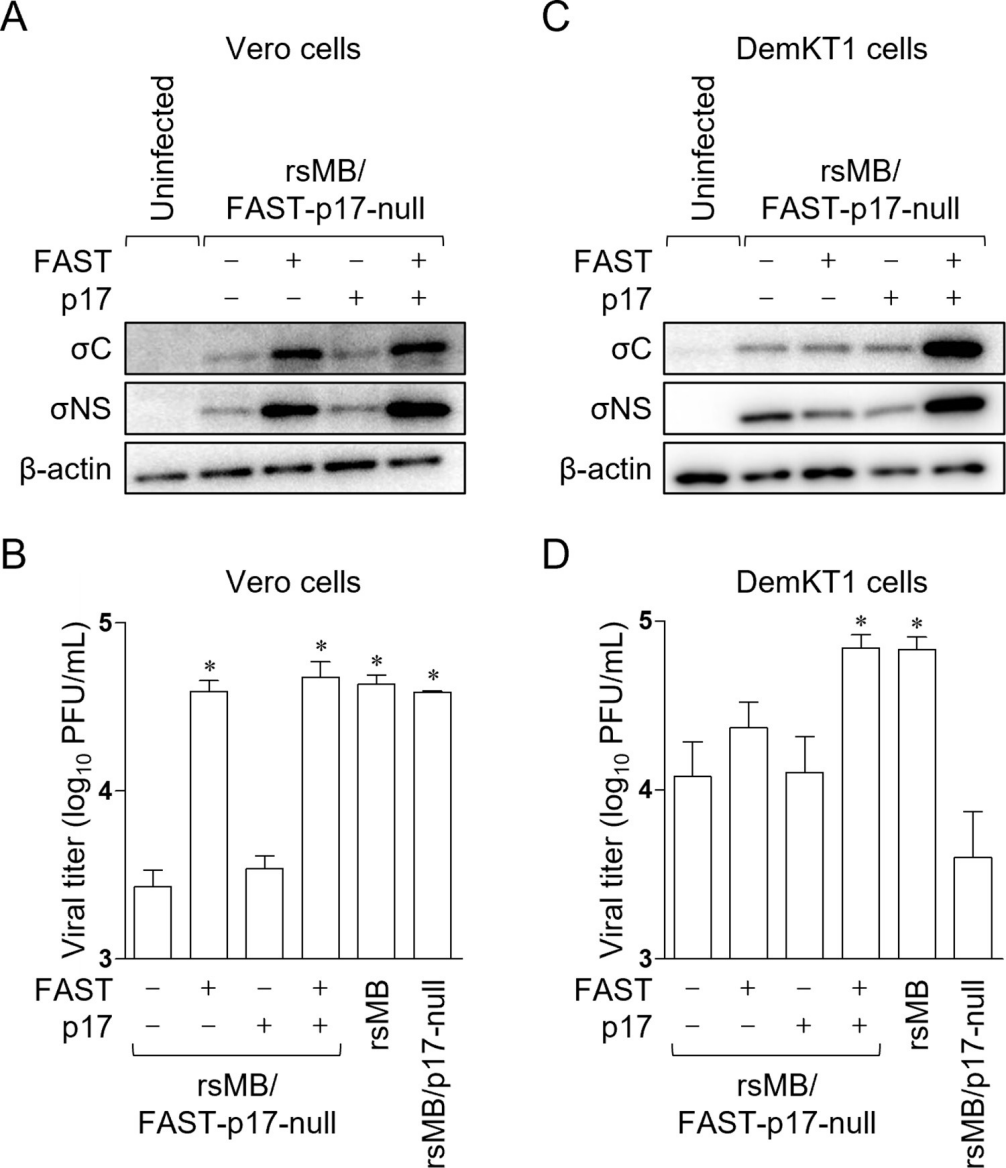
Efficient NBV replication requires both p17 and FAST in DemKT1 cells infected with rsMB/FAST-p17-null. **(A-D)** Complementation analysis of virus replication in cells infected with rsMB/FAST-p17-null in the presence of NBV p17 and/or NBV FAST proteins. NBV p17 and/or NBV FAST expression plasmids were transfected into Vero **(A, B)** and DemKT1 cells **(C, D)** infected with rsMB/FAST-p17-null at an MOI of 0.1 PFU/cell. Vero **(A)** and DemKT1 cells **(C)** were lysed at 12 and 21 h post-infection, respectively, and viral proteins were detected by immunoblotting with antibodies raised against NBV proteins or β-actin. Virus titers in infected Vero **(B)** and DemKT1 cells **(D)** were determined (n = 3) by plaque assay at 21 and 24 h post-infection, respectively. Results are expressed as the mean of three samples. Error bars indicate standard deviations. Significant differences compared to the negative control are indicated by asterisks. *p < 0.05 (Dunnett’s test).

To investigate the effects of the enhancement of FAST fusion activity by p17 on viral replication, we generated a recombinant virus that neither expressed FAST nor p17 (rsMB/FAST-p17-null) by disrupting the FAST start codon (^27^ATG^29^→ATT) and creating a stop codon (^39^TGC^41^→TGA) in an rsMB/p17-null background (S1 Table). A trans-complementation assay was then used to examine the effect of expression of p17 or FAST on viral replication. Vero and DemKT1 cells were transfected with expression plasmids encoding p17 and/or FAST prior to infection with rsMB/FAST-p17-null at an MOI of 0.1 and expression of viral proteins and titers in cells were determined. In Vero cells, σC and σNS expression and viral titers were restored by the expression of FAST, while viral protein expression and infectious titer were little affected by the restoration of p17 expression (Fig 8A and 8B), indicating that FAST protein alone is required for efficient viral replication in Vero cells, consistent with previous observations [37]. By contrast, co-expression of both FAST and p17 was required for restoration of viral protein expression and viral titers in DemKT1 cells (Fig 8C and 8D). Together, these data indicate that the enhancement of FAST function by p17 is essential for viral replication in DemKT1 cells.

### A p17 homologue from other bat-borne orthoreovirus enhances NBV replication in DemKT1 cells

**Fig 9:**
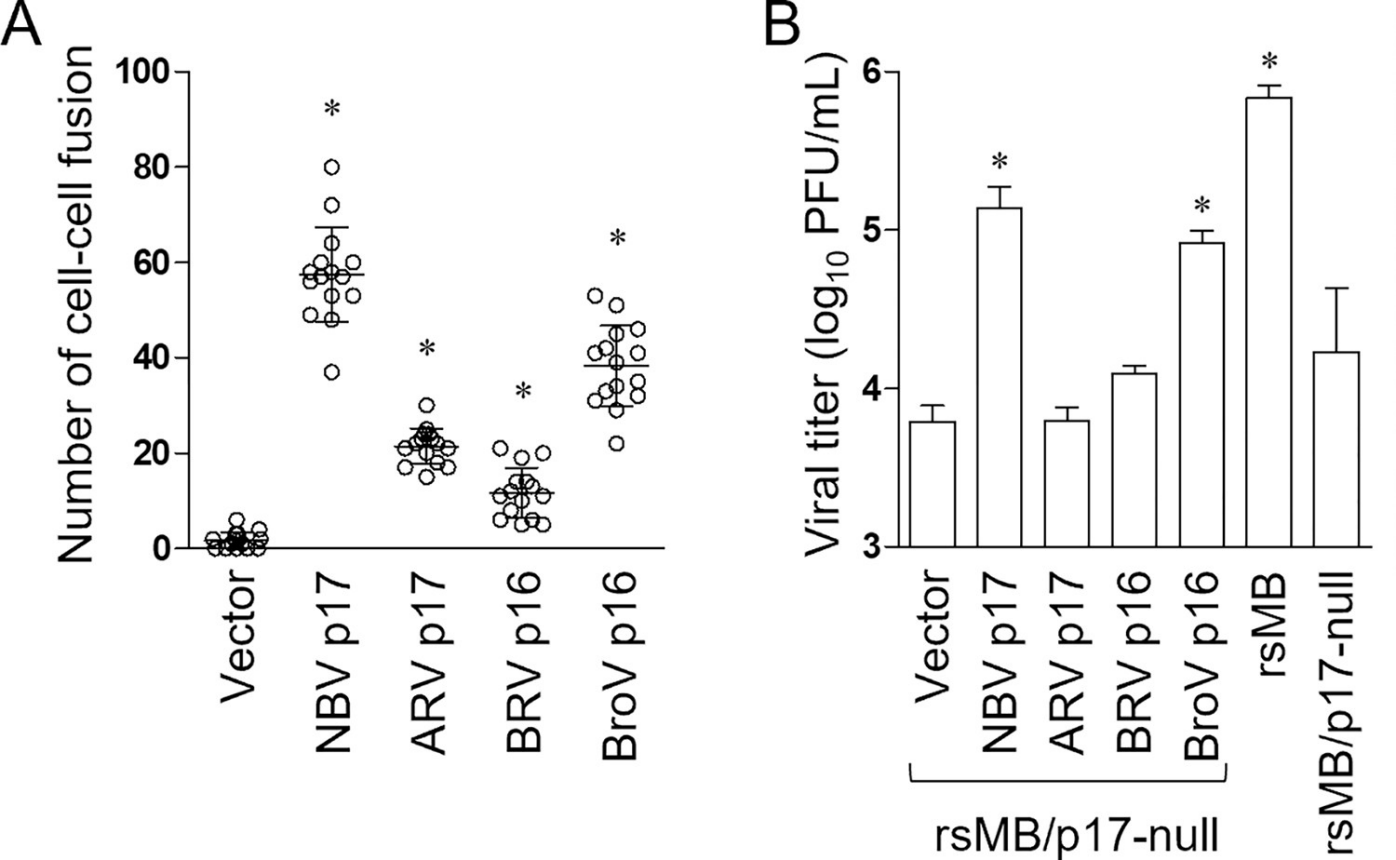
BroV p16 can restore rsMB/p17-null replication to complement cell–cell fusion activity in DemKT1 cells. **(A)** The number of cell–cell fusions in DemKT1 cells following NBV FAST co-expression with NBV p17, ARV p17, BRV p16, or BroV p16 in DemKT1 cells (n = 15). The number of fusion cells was counted in random microscopic fields (100×total magnification). **(B)** Viral titers of rsMB/FAST-p17-null in DemKT1 cells co-expressing NBV FAST with NBV p17, ARV p17, BRV p16, or BroV p16. Transfected cells were infected with rsMB/FAST-p17-null at an MOI of 0.1 PFU/cell (n = 6) at 3 h post-transfection. Viral titers were determined by plaque assay at 24 h post-infection. Results are expressed as the mean of total samples. Error bars indicate standard deviations. Significant differences compared to the vector control are indicated by asterisks. *p < 0.05 (Dunnett’s test).

Other fusogenic orthoreoviruses encode p17 homologues in gene segments encoding FAST proteins. ARV p17, for example, is encoded on the S1 gene segment, while the BRV and BroV p17 homologue, p16, is encoded on the S4 gene segment. To determine whether the p17 homologues have similar functions to NBV p17, we examined the activation of NBV FAST fusion activity in DemKT1 cells by co-expressing either NBV p17 or p17 homologues. ARV p17, BRV p16, and BroV p16 induced FAST-mediated cell–cell fusion and BroV p16, in particular, significantly increased cell–cell fusion (Fig 9A). We next performed complementation assays in DemKT1 cells infected with rsMB/p17-null using transient expression of the p17 homologues. We found that expression of BroV p16 but not of ARV p17 and BRV p16 restored viral replication in DemKT1 cells to a level comparable to that of wild-type NBV p17 (Fig 9B). Taken together, these data suggest that the regulation of NBV replication by the expression of p17 homologues in DemKT1 cells is virus species-specific.

## Discussion

The S1 gene segment of the bat-borne fusogenic virus NBV is tricistronic, encoding three partially overlapping open reading frames: FAST (27–314 nt), p17 (277–705 nt), and σC (572–1567 nt) [38]. Our previous studies have shown that NBV nonstructural FAST and structural σC proteins play a critical role in viral replication and pathogenesis [37,45]. In the present study, we found that while p17 does not contribute to NBV replication in many human and animal cell lines or to pathogenicity in mice (Figs 1 and 2), replication of a p17-deficient virus is remarkably impaired in cells derived from the natural host of NBV (Fig 3). Bats are the natural host for many highly pathogenic viruses, including SARS coronavirus and Nipah virus, but these viruses cause no clinical signs of disease in bats in general [12–15], suggesting that virus and host have co-evolved a benign relationship. It has been reported that bat immunology is unique, with their immune system able to repress and allow viral replication [6,16,51,52]. While most reports have focused on the host factors responsible for the regulation of infection, the viral factors responsible for viral replication in bats remain largely unknown and, therefore, the functional analysis of NBV p17 as a potential host-dependent factor influencing replication in bat cells is important for understanding the mechanism responsible for symbiosis between viruses and natural hosts.

Using various p17 truncation and substitution mutant viruses, we have identified basic amino acids at positions 117, 118, 131, and 132 that are critical for the enhancement of NBV FAST and the regulation of viral replication (Figs 5 and 6). These C-terminal amino acids are essential for the nuclear localization of NBV p17. Two residues, K117 and R118, are conserved between NBV and ARV strains, while downstream K131 and R132 residues are conserved among NBV strains only. Recently, the basic K122 and R123 residues of ARV p17 (K117 and R118 in NBV) were reported to act as a nuclear localization signal for the induction of autophagy, correlated with an enhancement in viral replication [40,53]. Thus, it is possible that NBV p17 may contribute to the regulation of viral replication by a similar pathway in bat cells. The downstream KR motif, which is conserved among NBV strains may be required for NBV-specific regulation of replication in bat cells.

We found that NBV p17 promotes viral replication by enhancing cell–cell fusion activity of FAST in bat cells (Figs 7 and 8). However, how NBV p17 enhances the fusogenic activity of FAST in bat cells is still unknown. It is possible that p17 affects host transcription and controls the expression of host factors that associate with FAST, as nuclear localization of p17 in bat cells is essential for its function. While it is unknown whether NBV p17 interacts with nucleic acids or cellular transcription factors, an interaction between ARV p17 and cellular heterogeneous nuclear ribonucleoprotein (hnRNP) A1 and lamin A/C has been reported and knockdown of these host factors inhibits the nucleocytoplasmic shuttling of ARV p17 [54]. We hypothesize that the interactions between NBV p17 and host factors such as hnRNP A1 and lamin A/C play a role in regulating nuclear localization of NBV p17 and directly or indirectly affect viral replication dependent on NBV p17 nuclear localization activity in bat cells.

A previous study reported that the cellular protein annexin A1 (AX1) interacts with reptilian orthoreovirus FAST-p14 protein and enhances cell–cell fusion in a calcium-dependent manner [55]. This interaction promotes membrane aggregation and is needed for fusion pore expansion mediated by FAST. AX1 similarly promotes syncytium formation by measles virus membrane fusion proteins F and H, suggesting that AX1 is necessary for syncytium formation. Thus, it is possible that NBV p17 may regulate expression and localization of host factors required for FAST-dependent cell–cell fusion such as AX1. Further studies are needed to understand which step of the cell–cell fusion process is regulated by p17 in bat cells. In the genus *Aquareovirus*, it is known that the Aquareovirus-C (AqRV-C) NS26 protein enhances the fusogenic activity of AqRV-C FAST-NS16 and co-localizes with lysosomes and lysosome-associated membrane protein 1 (LAMP1) to stimulate syncytium formation by FAST-NS16 [56,57]. The vacuolar-targeting TLPK motif of NS26 is critical for this function. Although the motif is not conserved in NBV p17 protein, it is possible that NBV p17 promotes the modification of lysosomes to enhance FAST-mediated fusion activity and viral replication in bat cells. Further analyses of cellular protein expression, metabolites and organelle function are required to improve our understanding of NBV p17 function in bat cells.

On the other hand, there is a possibility that bat DemKT1 cells encode unique restriction factors that inhibit the induction of cell–cell fusion by FAST. Previous studies have shown that several cellular restriction factors can inhibit the replication of viruses; apolipoprotein B mRNA-editing enzyme catalytic polypeptide-like 3G (APOBEC3G), for example, is efficiently incorporated into human immunodeficiency virus type 1 (HIV-1) particles, inhibiting viral replication by inducing G-to-A hypermutation in the viral cDNA during reverse transcription [58,59]. HIV-1 Vif protein can counteract this restriction by binding to APOBEC3G, leading to proteasomal degradation [58,60]. Tetherin, also known as BST-2, is a restriction factor that blocks the release of budded viral particles of retroviruses, such as HIV-1, and other enveloped viruses, including Ebola virus and vesicular stomatitis virus, by tethering virus particles to the plasma membrane [61–63]. Interestingly, the ability of these viral proteins to antagonize APOBEC3G and tetherin is species-specific [64–67]. Thus, it is possible that bat cells encode as-yet-unknown restriction factors associated with inhibition of FAST function that are disrupted by NBV p17.

NBV p17 enhances FAST function, regulating viral replication in a virus species-specific (Fig 9). Interestingly, a p17 homologue in another bat-borne orthoreovirus, BroV, efficiently enhances NBV FAST function in bat cells and is capable of restoring replication in p17-deficient NBV (Fig 9). ARV p17, which shares greater homology to NBV p17 than BroV p16, was incapable of restoring replication in p17-deficient viruses, suggesting potential co-evolution between fusogenic reoviruses and host species. While amino acid sequences differ, it is possible that the structures of NBV p17 and BroV p16 protein share similar characteristics. Further comparative studies regarding the structure and function of both proteins will promote understanding of fusogenic reovirus–host interactions. Bat-borne fusogenic reovirus p17 proteins have been suggested to control expression and/or localization of positive or negative host factors associated with FAST-dependent cell–cell fusion activity for efficient replication in bat cells. However, overexpression of NBV p17 promotes excessive FAST-mediated cell–cell fusion and viral replication, resulting in host cell damage. This would adversely affect both NBV and bats, suggesting that an as of yet unidentified mechanism between NBV and bats regulates the expression level of p17; for instance, the translation machinery of p17 could be the target of such a mechanism. NBV p17 is encoded by the tricistronic S1 gene segment and p17 is encoded as a second ORF between FAST and σC ORFs. A previous study demonstrated that leaky scanning is the predominant mechanism for ARV p17 translation from tricistronic S1 mRNA [68]. Such leaky scanning may affect the regulation of NBV p17 expression and play an important role in viral replication in bat cells. It is possible that other mechanisms could allow alternative bat-specific translation initiation of p17 by interacting or controlling host translational factors. The identification of bat factors interacting with NBV FAST and NBV p17, and understanding the translation mechanism of the three genes encoded by S1 gene segments, will be important for further analysis of the NBV replication regulation system in bats.

The present study shows that NBV p17 regulates viral replication in a host-specific manner. Further analysis of fusogenic reovirus p17 and FAST proteins in bat-borne viruses will contribute greatly to our understanding of the relationship between reservoir, virus, and the regulation of viral replication.

## Materials and methods

### Ethics statement

Animal studies were approved by the Animal Research Committee of the Research Institute for Microbial Diseases, Osaka University (Approval number: Bi-Dou-25-04-0). The experiments were conducted following the guidelines for the Care and Use of Laboratory Animals of the Ministry of Education, Culture, Sports, Science and Technology, Japan. The use of NBV strain MB was approved by the Research Institute for Microbial Diseases, Osaka University (Approval number: 24-Biken-362).

### Cell lines and viruses

The human cell lines A549, ACHN, ADR-RES, H23, OVCAR-3, OVCAR-4, OVCAR-5, SK-OV3, SK-MEL28, and T-47D were obtained from Dr. Toru Okamoto (the Walter & Eliza Hall Institute). Mouse fibroblast L929, mouse embryonic fibroblast (MEF), baby hamster kidney BHK, African green monkey kidney cell lines (Vero, CV-1, and MA104), canine kidney MDCK, and quail fibrosarcoma QT-6 cell lines were obtained from the American Type Culture Collection. Bovine kidney epithelial MDBK cell line and swine kidney SK-6 cell line [69] were kindly provided from Dr. Tomokazu Tamura. These human and animal cell lines were grown in Dulbecco’s modified Eagle’s medium (DMEM; Nacalai Tesque) supplemented with 5% fetal bovine serum (FBS; Gibco), 100 units/mL penicillin, and 100 μg/mL streptomycin (Nacalai Tesque). BHK-T7 cells were cultured in DMEM supplemented with 5% FBS, 100 units/mL penicillin, 100 μg/mL streptomycin, and 8 μg/mL puromycin (Sigma-Aldrich) [70]. DemKT1 and YubFKT1 cell lines were kindly provided from Dr. Ken Maeda [49] and grown in Roswell Park Memorial Institute 1640 medium (RPMI; Nacalai Tesque) supplemented with 5% FBS, 100 units/mL penicillin, and 100 μg/mL streptomycin. Wild-type rsMB was generated previously using a reverse genetics system [45]. Viruses were propagated in L929 cells. Viral titers were determined by plaque assay using L929 monolayers.

### Plasmid construction

NBV S1 rescue plasmids for the NBV reverse genetics system were generated using the plasmids pT7-MB-S1, pT7-NelB-S1, and pT7-Mel-S1 described elsewhere [45]. Name, mutation, and template plasmid used for the construction of all rescue plasmids are summarized in S1 Table. NBV MB p17 expression plasmid, pCXN2-NBV p17, was generated by cloning the MB p17 ORF into the pCXN2 vector. pCXN2-NBV p17-K131A, pCXN2-NBV p17-R132A, pCXN2-NBV p17-uKR-AA, pCXN2-NBV p17-dKR-AA, pCXN2-NBV p17-udKR-AA, and FLAG-tagged p17 expression plasmid (pCXN2-NBV p17-FLAG) were constructed from pCXN2-NBV p17. pCXN2-NBV p17-K131A-FLAG, pCXN2-NBV p17-R132A-FLAG, pCXN2-NBV p17-uKR-AA-FLAG, pCXN2-NBV p17-dKR-AA-FLAG, and pCXN2-NBV p17-udKR-AA-FLAG were constructed from pCXN2-NBV p17-FLAG. NBV MB FAST expression plasmid was generated by cloning MB FAST ORF into the pCAGGS vector (pCAG-NBV FAST). pCAG-NBV FAST-2HA was generated by adding two HA tags to the C-terminal end of FAST. To generate pCXN2-ARV p17, full-length p17 derived from ARV strain 58–132 [71] was amplified and cloned into the pCXN2 vector. BRV p16 (GenBank accession number: AF406787) and BroV p16 (GenBank accession number: GQ258986) were artificially synthesized (Eurofins Genomics). Nucleotide sequences of expression plasmids were confirmed by DNA sequencing.

### NBV reverse genetics system

A reverse genetics system employed for the NBV strain MB has been described previously [45]. BHK-T7 cells (1.5 × 10^5^) were seeded into 12-well plates (Corning) and transfected with 0.3 μg of each plasmid expressing an MB gene segment (pT7-MB-L1, pT7-MB-L2, pT7-MB-L3, pT7-MB-M1, pT7-MB-M2, pT7-MB-M3, pT7-MB-S1, pT7-MB-S2, pT7-MB-S3, and pT7-MB-S4) using 2 μL of TransIT-LT1 transfection reagent (Mirus) per μg of plasmid DNA. After incubation for 4–5 days, recombinant viruses were isolated from lysates by plaque purification using L929 cells.

### Electrophoretic analysis of dsRNA genomes

Viral genomic RNA was extracted from virions using Sepasol RNA I Super G (Nacalai Tesque) and mixed with an equal volume of 2 × sample buffer (125 mM Tris-HCl pH 6.8, 10% sucrose). The genomic RNA was separated using a 10% precast polyacrylamide gel (Atto) and visualized by ethidium bromide staining.

### Antibodies

Mouse monoclonal anti-β-actin (Sigma), rabbit polyclonal anti-lamin B1 (MBL), rabbit polyclonal anti-DDDDK-tag (FLAG tag; MBL), mouse monoclonal anti-HA-tag (Sigma), HRP conjugated anti-mouse IgG (Sigma), HRP conjugated anti-rabbit IgG (Sigma), CF488 goat anti-mouse IgG (Biotium), and CF594 goat anti-rabbit IgG (Biotium) antibodies were used for immunoblotting and immunofluorescence. To generate NBV σA, σC, σNS, and μNS antisera, ORFs expressing these proteins were cloned into the pTrcHisA vector (Life Technologies). Plasmids were transformed into the *E*. *coli* BL21 strain, and recombinant proteins were expressed and purified. The proteins were mixed with 2% Alhydrogel adjuvant (Invivogen) and subcutaneously inoculated into ICR mice (CLEA Japan). Anti-NBV p17 antiserum was obtained by rabbit immunization with 125–138 residues of NBV p17 (Sigma).

### Cell death assay

DemKT1 cells (3 × 10^4^) were plated in 96-well plates (Corning) and incubated overnight. The cells were infected with viruses at an MOI of 0.1 PFU/cell and incubated at 37°C for 1 h. Subsequently, the supernatant was removed, and the cells were washed with PBS and then incubated in RPMI (100 μL; 2% FBS) for various intervals. WST-1 (Roche) (20 μL) diluted to 10% in PBS was added to the cells and the cells were incubated at 37°C for 1 h. Cell viability was calculated by quantifying the cleavage of WST-1 to formazan by cellular succinate-tetrazolium reductase. Formazan was quantitated by measuring absorbance at A_450_nm-A_690_nm using a PowerScanHT (DS Pharma).

### Immunoblotting

Vero cells (1 × 10^5^) or DemKT1 cells (2.5 × 10^5^) were plated in 12-well plates and incubated overnight. Cells were infected with virus at an MOI of 0.1–3 PFU/cell and incubated at 37°C for 1 h. The supernatant was removed and cells were washed with PBS. The cells were incubated in DMEM or RPMI (2% FBS) for 16–20 h and cells were lysed in RIPA lysis buffer (25 mM Tris-HCl pH 7.4, 150 mM NaCl, 1% sodium deoxycholate, 0.1% SDS). Lysates were mixed with 2×sample buffer (125 mM Tris-HCl pH 6.8, 10% 2-mercaptoethanol, 4% SDS, 10% sucrose), separated on a 10%, 12%, or 14% polyacrylamide gels, and blotted onto polyvinylidene difluoride membranes (Millipore). The samples were incubated in primary and secondary antibodies, and proteins were detected using Chemi-Lumi One Ultra (Nacalai Tesque) and LAS-4000 (Fujifilm) or Amersham Imager 600UV (GE Healthcare).

### Infection of mice

Four-week-old male C3H mice (CLEA, Japan) were intranasally infected with 20 μL of viruses diluted in PBS (3×10^5^ PFU). Mice were euthanized when moribund.

### Immunofluorescence analysis

p17 expression plasmids were transfected into DemKT1 cells (1×10^5^) on glass coverslips. At 25 h post-transfection, samples were fixed with 4% paraformaldehyde and permeabilized using 0.5% Triton-X. DemKT1 cells (2×10^5^) and Vero cells (1×10^5^) were infected with virus at an MOI of 0.1 PFU/cell at 37°C for 1 h, and cells were washed and incubated in growth media. At 13–26 h post-infection, samples were fixed with 4% paraformaldehyde and permeabilized using 0.5% Triton-X. Fixed cells were incubated with antibodies and DAPI or Hoechst, and the samples were mounted using ProLong diamond antifade mountant (Thermo Fisher scientific). Images were acquired with a FluoView FV1000 laser scanning confocal microscope (Olympus).

### Subcellular fractionation

pCXN2-NBV p17-FLAG or p17 mutant plasmids were transfected into DemKT1 cells (3×10^5^). At 24 h post-transfection, cells were washed with PBS and collected by trypsin digestion. The cells were resuspended in suspension buffer (10 mM HEPES-KOH (pH 7.9), 10 mM KCl, 1.5 mM MgCl_2_, and 2.5% mercaptoethanol) and collected by centrifugation (6,000×g, 5 min, 4°C). The cells were subsequently lysed in 200 μL of suspension buffer supplemented with 1.25% NP-40 (Nacalai Tesque) on ice, vortexing every minute for 5 min. Samples were centrifugated at 6,000×g for 15 min at 4°C and the supernatant was collected as the cytoplasmic fraction. The pellet was washed with suspension buffer 3 times and lysed in 200 μL of RIPA lysis buffer. The lysed pellet was centrifugated at 6,000×g for 5 min at 4°C and the supernatant was collected as the nuclear fraction.

### Cell–cell fusion assay

Cells were plated at an appropriate concentration (DemKT1 cells at 4 × 10^5^ cells/well and Vero cells at 2 × 10^5^ cells/well in 12-well plates; YubFKT1 cells at 1 × 10^5^ cells/well in 24-well plates) and transfected with p17 and/or FAST expression vectors. At 16–26 h post-transfection, cells were fixed with methanol and stained with Giemsa’s Stain Solution (Nacalai Tesque), and the number of fusion cells were counted in random microscopic fields (100×total magnification).

### Complementation of viral replication in cells infected with a p17 mutant virus

To examine viral protein expression, pCXN2-NBV p17 (1 μg) was transfected into DemKT1 cells (2.5 × 10^5^) and, at 10 h post-transfection, infected with rsMB/p17-null at an MOI of 1 PFU/cell. After 1 h, the cells were washed with PBS and incubated in growth media for 16 h at 37°C. The cells were lysed and viral proteins were detected using antibodies against NBV proteins. To examine viral replication, pCXN2-NBV p17 (0.5 μg) was transfected into DemKT1 cells (5 × 10^4^) and, at 24 h post-transfection, infected with rsMB/p17-null at an MOI of 0.1 PFU/cell. After 1 h, the cells were washed with PBS and incubated in growth media for 24 h at 37°C. The cells were collected and viral titers were determined by plaque assay on a monolayer of L929 cells.

### Complementation of viral replication in cells infected with a FAST-p17 mutant virus

To examine viral protein expression, pCXN2-NBV p17 (1 μg) was transfected into DemKT1 cells (2.5 × 10^5^) or Vero cells (1 × 10^5^) with or without pCAG-NBV FAST (1 μg). At 3 h post-transfection, the DemKT1 cells were infected with rsMB/FAST-p17-null at an MOI of 0.1 PFU/cell. After 4 h of incubation, the cells were washed with PBS and incubated for 17 h at 37°C. The Vero cells were infected with the virus at an MOI of 0.1 PFU/cell at the same time of transfection, incubated for 12 h at 37°C, and lysed. Viral proteins were detected using antibodies against NBV proteins. To examine viral replication, pCXN2-NBV p17 (0.5–1.0 μg) was transfected into DemKT1 cells (1 × 10^5^) or Vero cells (5 × 10^4^) with or without pCAG-NBV FAST (0.5–1.0 μg). At 3 h post-transfection, the cells were infected with rsMB/FAST-p17-null at an MOI of 0.1 PFU/cell. After 1 h of incubation, the cells were washed with PBS and incubated for 17–20 h at 37°C. The supernatant was collected and viral titers were determined by plaque assay on a monolayer of L929 cells.

### Statistical analysis

Each data point is expressed as the mean of at least three samples and error bars indicate the standard deviation. Statistical significance was determined via Welch’s *t*-test, log rank test, or one-way ANOVA followed by Dunnett’s test where indicated. p values < 0.01 or 0.05 were considered statistically significant.

## Supporting information

S1 Fig Survival rates of DemKT1 cells infected with wild-type rsMB or rsMB/p17-null viruses. DemKT1 cells were infected with rsMB or rsMB/p17-null at an MOI of 0.1 PFU/cell (n = 3). The survival rate was determined by measuring succinate-tetrazolium reductase activity. Results are expressed as the mean of the results of all samples. Error bars indicate standard deviations.
(TIF)

S2 Fig Overexpression of FAST alone cannot induce cell–cell fusion in DemKT1 cells. **(A)** FLAG-tagged NBV p17 expression plasmids and/or the HA-tagged NBV FAST expression plasmid were transfected into DemKT1 cells (4 × 10^5^ cells). At 26.5 h post-transfection, the samples were collected. Protein expression was detected by immunoblotting. **(B)** The number of cell–cell fusions in DemKT1 cells expressing FLAG-tagged NBV p17 and/or HA-tagged NBV FAST. FLAG-tagged NBV p17 expression plasmids and/or the HA-tagged NBV FAST expression plasmid were transfected into DemKT1 cells (4 × 10^5^ cells). At 26.5 h post-transfection, the cells were fixed and stained with Giemsa’s Staining Solution. The number of fusion cells was counted in random microscopic fields (100×total magnification). Results are expressed as the mean of the results of all samples. ND, not detected. Error bars indicate standard deviations (n = 5).
(TIF)

S3 Fig Cell–cell fusion activity of FAST was enhanced by p17 in YubFKT1 cells. The number of cell–cell fusion events in YubFKT1 cells transfected with NBV p17 and/or FAST expression plasmids at 24 h post-transfection. The number of fusion cells was counted in random microscopic fields (100×total magnification). Results are expressed as the mean of the results of all samples. Error bars indicate standard deviations (n = 8). Significant differences compared to the vector control are indicated by asterisks. *p < 0.05 (Dunnett’s test).
(TIF)

S4 Fig Induction of cell-cell fusion by FAST is mediated by FLAG-tagged p17 proteins. **(A)** The number of cell–cell fusions in DemKT1 cells expressing NBV FAST and FLAG-tagged wild-type or mutant p17s. The number of fusion cells was counted in random microscopic fields (100×total magnification). Results are expressed as the mean of the results of all samples. ND, not detected. Error bars indicate standard deviations (n = 5). **(B)** Expression levels of p17 and FAST in transfected DemKT1 cells. FLAG-tagged NBV p17 expression plasmid (0.5 μg) was transfected with the NBV FAST expression plasmid (0.5 μg) into DemKT1 cells (4 × 10^5^ cells). At 18 h post-transfection, the samples were collected. Protein expression was detected by immunoblotting.
(TIF)

S1 Table Amino acid mutations of FAST, p17, and σC in recombinant viruses. (TIF)
