# Supplementary figures and images for: The nonstructural p17 protein of a fusogenic bat-borne reovirus regulates viral replication in virus species- and host-specific manners
Source: PLoS Pathog. 2022 Jun 2;18(6):e1010553. doi: 10.1371/journal.ppat.1010553 (PMC9162341; doi:10.1371/journal.ppat.1010553)

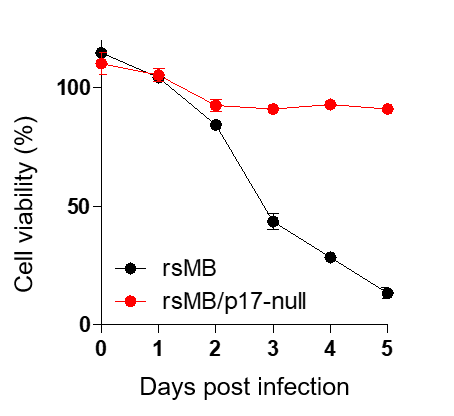

Supplement: S1 Fig — DemKT1 cells were infected with rsMB or rsMB/p17-null at an MOI of 0.1 PFU/cell (n = 3). The survival rate was determined by measuring succinate-tetrazolium reductase activity. Results are expressed as the mean of the results of all samples. Error bars indicate standard deviations. (TIF) [file ppat.1010553.s001.tif]

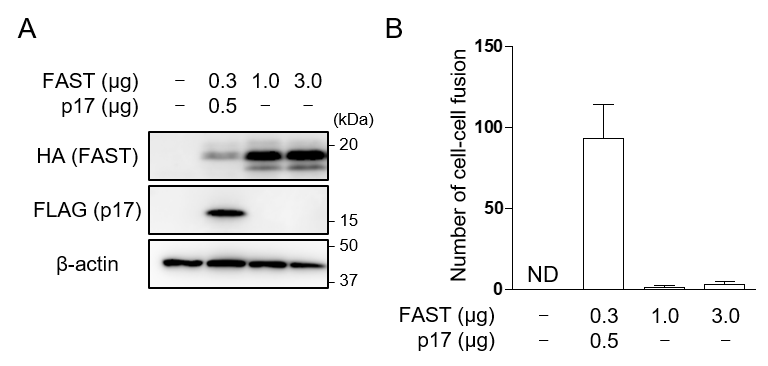

Supplement: S2 Fig — (A) FLAG-tagged NBV p17 expression plasmids and/or the HA-tagged NBV FAST expression plasmid were transfected into DemKT1 cells (4 × 10^5^ cells). At 26.5 h post-transfection, the samples were collected. Protein expression was detected by immunoblotting. (B) The number of cell–cell fusions in DemKT1 cells expressing FLAG-tagged NBV p17 and/or HA-tagged NBV FAST. FLAG-tagged NBV p17 expression plasmids and/or the HA-tagged NBV FAST expression plasmid were transfected into DemKT1 cells (4 × 10^5^ cells). At 26.5 h post-transfection, the cells were fixed and stained with Giemsa’s Staining Solution. The number of fusion cells was counted in random microscopic fields (100×total magnification). Results are expressed as the mean of the results of all samples. ND, not detected. Error bars indicate standard deviations (n = 5). (TIF) [file ppat.1010553.s002.tif]

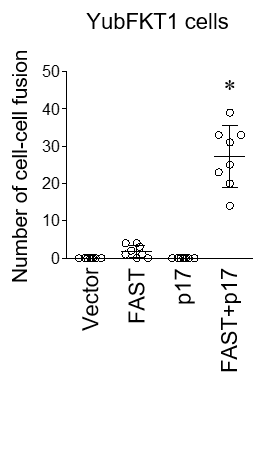

Supplement: S3 Fig — The number of cell–cell fusion events in YubFKT1 cells transfected with NBV p17 and/or FAST expression plasmids at 24 h post-transfection. The number of fusion cells was counted in random microscopic fields (100×total magnification). Results are expressed as the mean of the results of all samples. Error bars indicate standard deviations (n = 8). Significant differences compared to the vector control are indicated by asterisks. *p < 0.05 (Dunnett’s test). (TIF) [file ppat.1010553.s003.tif]

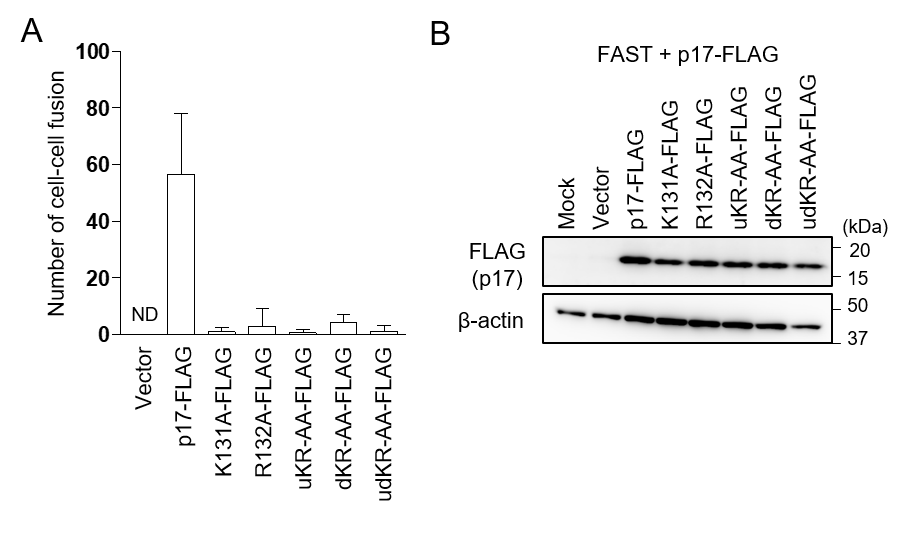

Supplement: S4 Fig — (A) The number of cell–cell fusions in DemKT1 cells expressing NBV FAST and FLAG-tagged wild-type or mutant p17s. The number of fusion cells was counted in random microscopic fields (100×total magnification). Results are expressed as the mean of the results of all samples. ND, not detected. Error bars indicate standard deviations (n = 5). (B) Expression levels of p17 and FAST in transfected DemKT1 cells. FLAG-tagged NBV p17 expression plasmid (0.5 μg) was transfected with the NBV FAST expression plasmid (0.5 μg) into DemKT1 cells (4 × 10^5^ cells). At 18 h post-transfection, the samples were collected. Protein expression was detected by immunoblotting. (TIF) [file ppat.1010553.s004.tif]

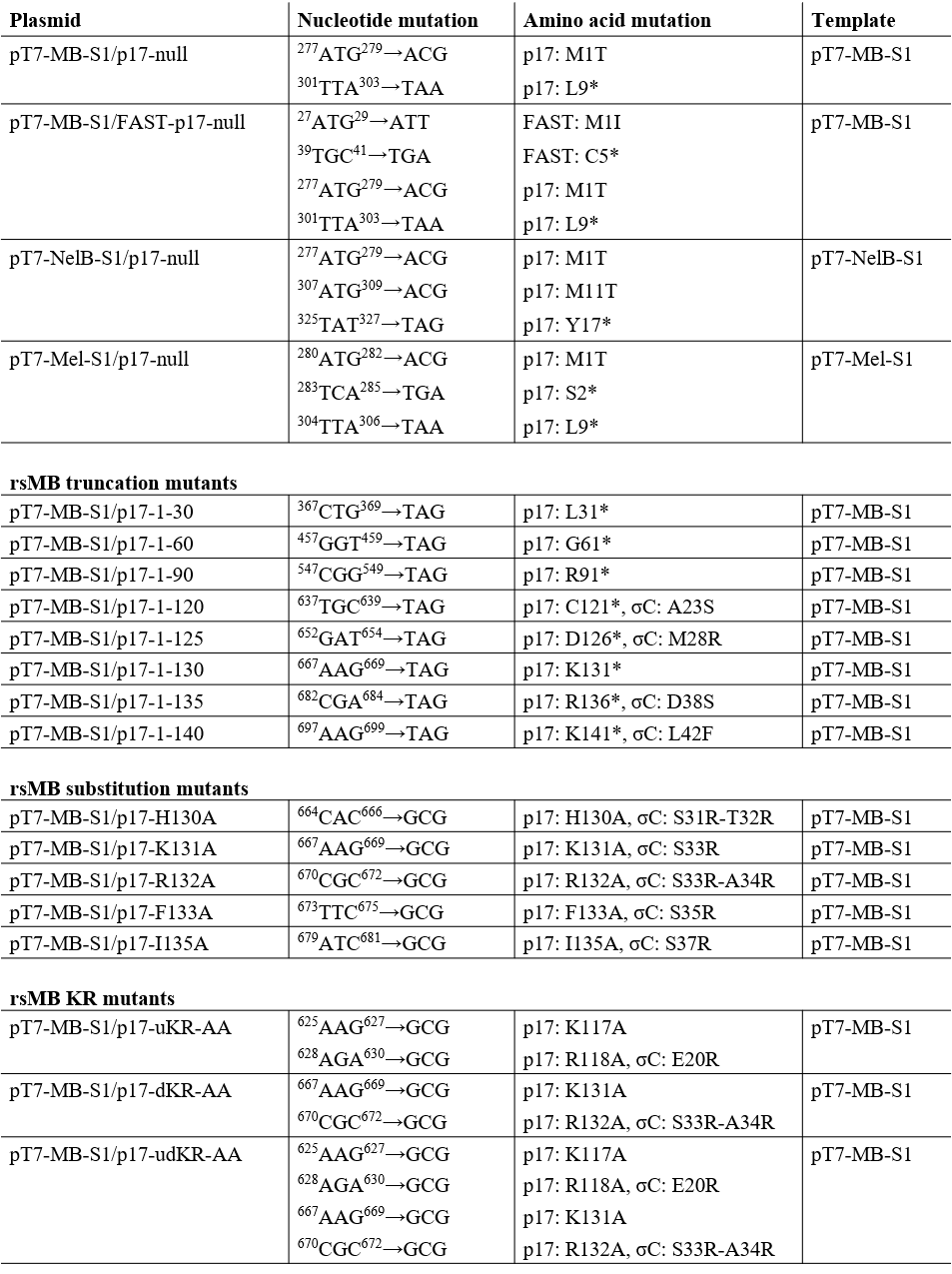

Supplement: S1 Table — (TIF) [file ppat.1010553.s005.tif]
